# Resveratrol Supplementation Modulates Endothelial Dysfunction in Alzheimer’s Disease (AD): *In Vitro* Effects on Human Aortic Endothelial Cells Exposed to AD Plasma

**DOI:** 10.2174/011570159X382096250914055513

**Published:** 2025-10-06

**Authors:** Valentina Schiavoni, Sonila Alia, Veronica Pompei, Chiara Fiori, Simona Luzzi, Arianna Vignini, Eleonora Salvolini, Mauro Silvestrini, Monica Emanuelli

**Affiliations:** 1 Department of Clinical Sciences, Polytechnic University of Marche, Via Tronto 10/A, Ancona, 60126, Italy;; 2 Department of Experimental and Clinical Medicine, Polytechnic University of Marche, Via Conca 71, Ancona, 60126, Italy

**Keywords:** Alzheimer’s disease, endothelial cells, membrane fluidity, Na^+^/K^+^-ATPase, nitric oxide, peroxynitrite, oxidative stress, resveratrol, superoxide dismutase

## Abstract

**Introduction:**

Increasing evidence indicates a connection between Alzheimer’s disease (AD) and endothelial dysfunction. Given the lack of a definitive cure for AD, the purpose of this research was to explore the impact of a short incubation with plasma samples obtained from 30 patients with sporadic AD and 21 age- and sex-matched control subjects on cultured human aortic endothelial cells (HAECs), as well as to assess the effects of resveratrol (RSV) supplementation to the plasma.

**Methods:**

Specifically, the study analyzed: the production of nitric oxide (NO) and peroxynitrite; the activities of superoxide dismutase (SOD) and Na^+^/K^+^-ATPase; membrane fluidity; and levels of thiobarbituric acid-reactive substances (TBARS).

**Results:**

When incubated with AD plasma, cells showed a decrease in NO levels, enzymatic activities, and membrane fluidity, as well as an increase in peroxynitrite and TBARS production, compared to those exposed to plasma from healthy controls. In contrast, supplementation with RSV-enriched plasma, reduced reactive oxygen species (ROS) levels, and enhanced SOD activity. RSV also improved endothelial function, by increasing membrane fluidity, Na^+/^K^+^-ATPase activity, and enhancing NO production and bioavailability, potentially benefiting cerebral perfusion.

**Discussion:**

Though preliminary, our findings highlight the critical role played by vascular health in Alzheimer’s disease, and the potential impact of resveratrol in maintaining the endothelial integrity, thus mitigating the progression of AD .

**Conclusion:**

In conclusion, our study supports the use of dietary natural compounds to reduce oxidative stress and prevent or reverse vascular endothelial dysfunction associated with AD.

## 1 INTRODUCTION

Alzheimer’s disease (AD) is a progressive neurodegenerative disorder responsible for 60-80% of dementia cases [1]. As life expectancy increases, so does the prevalence of AD, putting growing pressure on the healthcare system [2]. Although AD is traditionally recognized by the widespread accumulation of amyloid β (Aβ) plaques and the aggregation of hyperphosphorylated tau protein into neurofibrillary tangles within neurons, it is not merely a disorder of the Central Nervous System (CNS) [3]. Increasing evidence suggests that vascular involvement plays a critical role in AD pathogenesis. According to this hypothesis, neurodegeneration begins with chronic cerebral hypoperfusion, which may be driven by aging or vascular conditions like hypertension, atherosclerosis, and type II diabetes [4, 5]. This reduced blood flow can alter the structure of cerebral capillary, disrupting microcirculation and impairing the supply of oxygen and glucose to brain cells and the blood-brain barrier (BBB), ultimately contributing to neurodegeneration [6]. Furthermore, this microvascular dysfunction impairs the clearance of Aβ, leading to its accumulation in the extracellular space [4]. Studies have also shown a link between brain microvascular abnormalities and Aβ deposition [7]. Additionally, BBB impairment can disrupt nitric oxide (NO) production by endothelial cells, further decreasing regional blood flow and accelerating neurodegeneration [4]. NO, a multifunctional free radical, is produced from L-arginine by three isoforms of nitric oxide synthase (NOS): endothelial (eNOS), neuronal (nNOS), and inducible (iNOS), which is generally expressed in response to inflammatory cytokines and growth factors and releases high levels of NO. NO produced by eNOS acts as a vasodilator, playing a key role in regulating blood flow [8]. Reduced NO production may also result from an increase in circulating Aβ, which can accumulate on the vascular endothelium and inhibit eNOS activity [9]. Impaired cerebral perfusion could be driven not only by decreased NO production but also by a loss of NO bioavailability due to its interaction with superoxide anions, forming peroxynitrite—a powerful oxidizing and nitrating agent that damages nucleic acids, lipids, and proteins [10, 11]. This hypothesis is supported by observations of elevated reactive oxygen species (ROS) levels in both the brains and plasma of AD patients [12, 13] as well as a significant reduction in antioxidant enzyme activity [14]. The resulting oxidative stress can lead to eNOS uncoupling, causing superoxide production instead of NO, which in turn further exacerbates oxidative stress and disrupts cell membrane fluidity, potentially impairing the function of membrane-bound enzymes like Na^+^/K^+^-ATPase [15, 16]. ROS accumulation can also directly damage Na^+^/K^+^ pump subunits [17]. Considering that endothelial dysfunction occurs early in AD progression, we previously investigated how a short incubation with plasma from mild cognitive impairment (MCI) and AD patients affects cultured human aortic endothelial cells (HAECs) [18]. We specifically measured NO production, superoxide dismutase (SOD) activity, Na^+^/K^+^-ATPase activity, membrane fluidity, and the levels of thiobarbituric acid-reactive substances (TBARS) as a marker of lipid peroxidation. Building on these results, in the current study, we evaluated the same parameters, along with peroxynitrite formation, following incubation with AD plasma. We also repeated these measurements after incubating the plasma supplemented with resveratrol (RSV), a polyphenolic phytoalexin found in various plant species known for its antioxidant and anti-inflammatory properties [19-22]. Notably, previous research has highlighted that resveratrol may help protect against memory decline in AD [23].

## 2 MATERIALS AND METHODS

### 2.1 Patients

The study was approved by the Institutional Review Board of the Polytechnic University of Marche, and adhered to the principles outlined in the Declaration of Helsinki, as revised in 2001. A total of 30 AD patients (17 women and 13 men, aged 75-86 years) and 21 age- and sex-matched healthy controls (11 women and 10 men) participated in the study.

To justify the adequacy of the sample size for achieving reliable and statistically significant results, a power analysis was performed using a one-way ANOVA. The calculation was based on data from our previous study [18]. Using the observed difference in concentration values and variability reported in that study, we estimated that the inclusion of 30 patients and 21 controls would provide at least 80% power to detect a statistically significant difference between groups at a significance level of 0.05. AD diagnoses were made based on international criteria [24]. Participants with acute comorbidities (*i.e*, stroke, head trauma, delirium) were excluded. In order to ensure a homogeneous sample, only patients with sporadic AD were included; therefore, the sample did not include subjects with hereditary or early-onset AD. All participants, or their caregivers, provided written informed consent prior to enrolment.

### 2.2 Plasma Separation

Peripheral blood was collected after an overnight fast into heparinized tubes. Plasma was separated by centrifugation at 3000 rpm for 10 minutes at 4°C and stored at -80°C until further analyses.

### 2.3 Human Aortic Endothelial Cell (HAEC) Culture

HAECs were purchased from Lonza (Lonza Group Ltd, Basel, Switzerland) and cultured as previously described [18]. When cells reached 80% confluence, they were incubated for 3 hours at 37°C with 20% (v/v) plasma in EGM-2 (HAEC + C plasma from healthy controls, HAEC + AD plasma from AD patients) or with plasma supplemented with resveratrol (RSV) to a final concentration of 20 µg/ml (HAEC + C plasma + RSV, HAEC + AD plasma + RSV) [25]. The incubation time was chosen based on previous *in vitro* studies that showed short-term exposure can influence vascular tone [9]. Following incubation, the supernatants were collected to measure NO release and peroxynitrite formation, while cells were harvested by gentle scraping for the analysis of SOD and Na^+^/K^+^-ATPase activities, membrane fluidity, and TBARS.

### 2.4 NO Production

A commercially available kit (Total Nitric Oxide Assay Kit, Stressgen; Assay Designs, Inc., Ann Arbor, MI, USA) was used to measure NO release from HAECs. The assay converts all nitrate in the sample to nitrite, which is then quantified. NO levels were determined using a standard curve and measured at 540 nm with a microplate reader (Multiskan GO, Thermo Fisher Scientific, Inc., Waltham, MA, USA). The amount of NO in each specimen was expressed as nmol/ml.

### 2.5 Preparation of 2,7-dichlorofluorescein Diacetate (DCFDA)-free Base

DCFDA-free base solution was prepared by mixing 0.05 ml of 10 mM/l DCFDA with 2 ml of 0.01 N NaOH, at room temperature for 30 min. The mixture was then neutralized with 18.0 ml of 25 mmol/l phosphate-buffered saline (PBS), pH 7.4 This solution was maintained on ice in the dark until use [26].

### 2.6 Peroxynitrite Production

Peroxynitrite (ONOO^-^) production was evaluated using the fluorescent probe DCFDA, as previously described [27]. In brief, samples were incubated with 5 μM DCFDA-free base at 37°C for 15 minutes. Following this, the samples were treated with or without L-arginine (100 mM) and NG-monomethyl-L-arginine (L-NMMA) (100 mM) for an additional 15 minutes at 37°C in the dark. After washing with PBS (pH 7.4), cells were lysed by sonication. The resulting mixture was centrifuged at 1000 rpm for 5 minutes, and the fluorescence in the supernatant was measured using a PerkinElmer LS-50B spectrofluorometer (PerkinElmer, Inc., Waltham, MA, USA), with an excitation wavelength of 475 nm and emission at 520 nm.

### 2.7 SOD and Na^+^/K^+^-ATPase Activity

SOD activity was quantified using a commercial kit (Superoxide Dismutase Activity Kit, Stressgen, Assay Designs Inc., Ann Arbor, MI, USA) and expressed as U/mg protein. Na^+^/K^+^-activated Mg^2+^-dependent ATPase activity was assessed according to the method of Kitao and Hattori [28], as detailed earlier [29]. To calculate Na^+^/K^+^-ATPase activity, the ATPase activity measured in the presence of 10 mM ouabain was subtracted from the total Mg^2+^-dependent ATPase activity, with the final value expressed as micromoles of inorganic phosphate (P_i_) per milligram of protein per hour (μmol P_i_/mg protein/h). Protein concentration was quantified using the Bradford BioRad protein assay, with serum albumin as the standard [30].

### 2.8 Membrane Fluidity

HAEC membrane fluidity was evaluated by determining the fluorescence anisotropy (reciprocal of fluidity) of probes 1-(4-trimethylammoniophenyl)-6-phenyl-1,3,5-hexatriene (TMA-DPH), which is incorporated into the lipid-water interface of the membrane bilayer, and 1-6-phenyl-1,3,5-hexatriene (DPH), which is a completely hydrophobic fluorescent probe. Cells were incubated with DPH and TMA-DPH as outlined previously [29]. Fluorescence intensities (100 readings each) of both vertical and horizontal emitted light components were measured using a Perkin-Elmer MPF66 spectrofluorometer (PerkinElmer, Inc., Waltham, MA, USA) equipped with two glass prism polarizers, at an emission wavelength of 430 nm and at an excitation wavelength of 365 nm. The steady-state fluorescence anisotropy (r) of TMA-DPH and DPH was determined using the equation provided below:

r = (I_v_G – I_h_) / (I_v_ + 2I_h_)

where G represents the instrumental factor that corrects the r value for unequal detection of vertically (I_v_) and horizontally (I_h_) polarized light. The sample temperature was kept constant at 37°C by an external bath circulator (Haake F3).

### 2.9 Thiobarbituric Acid Reactive Substances (TBARS)

Oxidative stress leads to the generation of highly reactive lipid hydroperoxides, which are derived from polyunsaturated fatty acids. These hydroperoxides are unstable, and their breakdown produces malondialdehyde (MDA), which can be quantified colorimetrically through its reaction with thiobarbituric acid (TBA). TBARS levels were assessed using a commercial kit (TBARS Assay kit, Cayman Chemical Company, Ann Arbor, MI, USA) and were expressed as nmol/mg prot.

### 2.10 Statistical Analysis

Statistical analysis was performed using the SAS statistical package (Statistical Analysis System Institute, Cary, NC, USA). Each experiment was run in triplicate and independently repeated three times. Results were expressed as mean ± standard deviation (SD). Differences between groups were assessed by analysis of variance (ANOVA), followed by a Bonferroni t-test to reduce the probability of significant differences arising by chance. Differences with *p* < 0.05 were considered statistically significant.

## 3 RESULTS

**Fig. (1):**
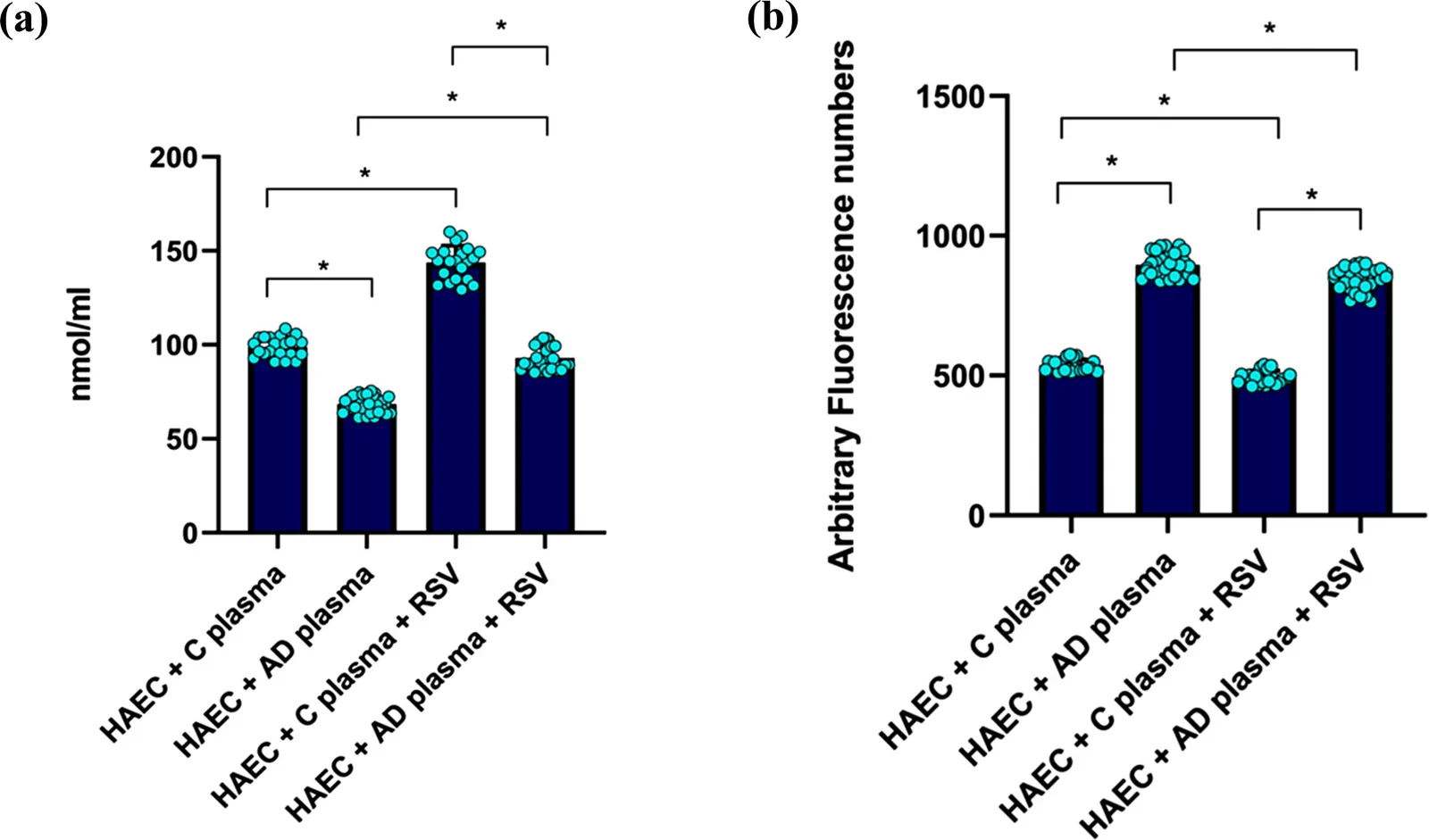
(**a**) NO release and (**b**) peroxynitrite production in HAECs after incubation with control plasma (HAEC + C plasma), plasma from AD patients (HAEC + AD plasma), control plasma supplemented with resveratrol (HAEC + C plasma + RSV), and AD plasma supplemented with resveratrol (HAEC + AD plasma + RSV). Means ± S.D. and individual data points are shown. *p < 0.05.

The effects of incubating HAECs with plasma from AD patients and healthy subjects both before and after resveratrol supplementation, were examined in this study. NO and peroxynitrite production, SOD and Na^+^/K^+^-ATPase activities, membrane fluidity, and TBARS levels were specifically measured. NO production was significantly reduced in HAECs incubated with AD plasma with respect to cells exposed to plasma from healthy controls. Conversely, RSV supplementation resulted in a significant increase in NO production in both groups (HAEC + C plasma = 98.76 ± 5.37 nmol/ml; HAEC + AD plasma = 68.22 ± 4.41 nmol/ml; *p* < 0.05). After RSV treatment, NO levels significantly increased (HAEC + C plasma + RSV = 143.77 ± 8.99 nmol/ml; HAEC + AD plasma + RSV = 92.89 ± 6.23 nmol/ml; *p* < 0.05) (Fig. **1a**). In addition, endothelial cells incubated with plasma from Alzheimer's disease patients exhibited a significantly higher level of peroxynitrite formation than those exposed to plasma from control individuals. On the contrary, significantly lower levels were found in both groups after the addition of RSV (being HAEC + C plasma = 535.70 ± 21.08; HAEC + AD plasma = 895.39 ± 41.27; HAEC + C + RSV = 493 ± 23.38; HAEC + AD plasma + RSV = 845.78 ± 39.91; *p* < 0.05) (Fig. **1b**).

**Fig. (2):**
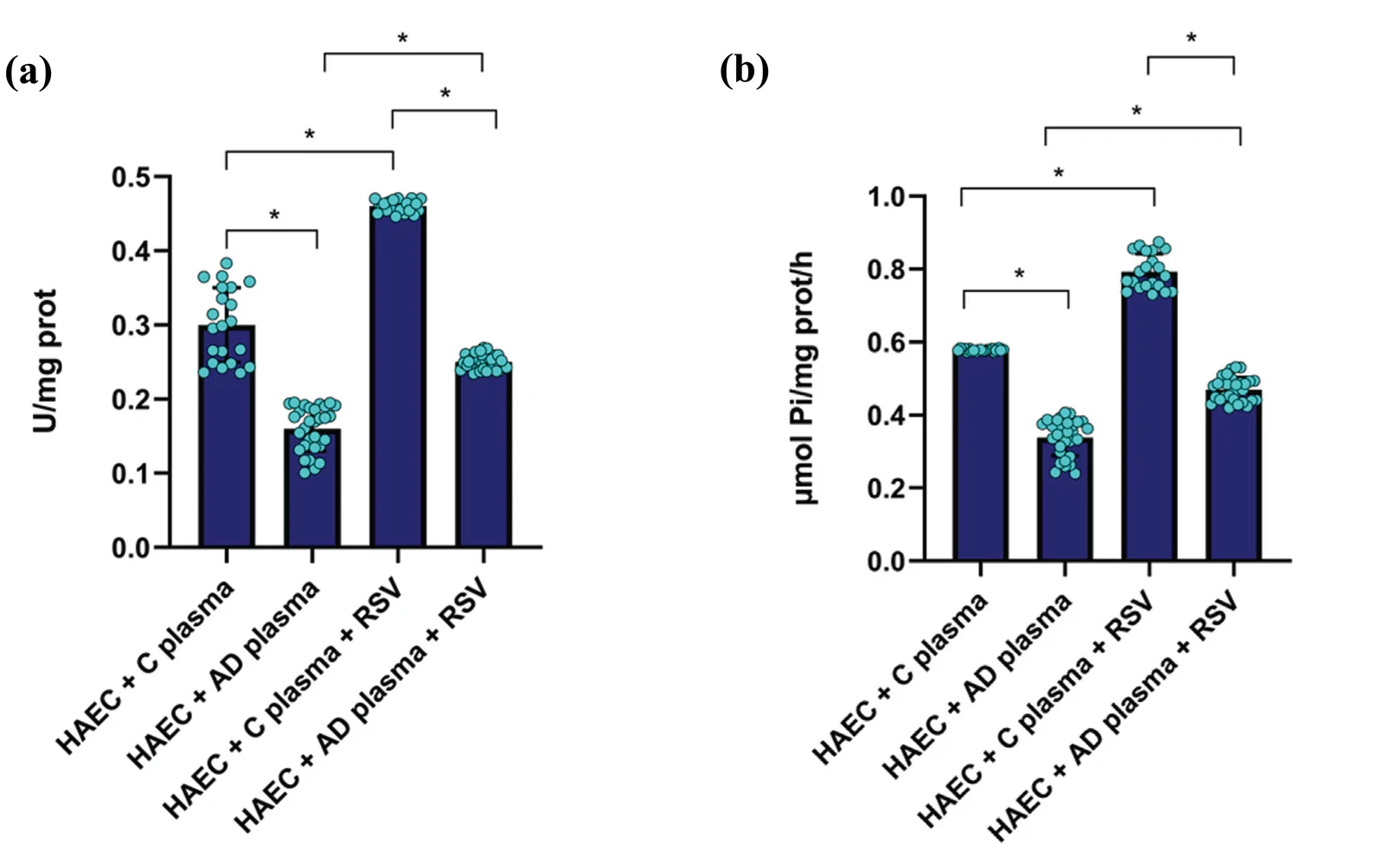
(**a**) SOD and (**b**) Na^+^/K^+^ - ATPase activities in HAECs following incubation with plasma from healthy subjects (HAEC + C plasma), AD plasma (HAEC + AD plasma), control plasma supplemented with resveratrol (HAEC + C plasma + RSV), and plasma from AD patients supplemented with resveratrol (HAEC + AD plasma + RSV). Means ± S.D. and individual data points are shown. *p < 0.05.

A similar pattern was observed for enzymatic activity. Both SOD and Na^+^/K^+^ - ATPase were significantly lower in HAECs exposed to AD plasma compared to control plasma. RSV supplementation significantly increased the activity of both enzymes (being SOD: HAEC + C plasma = 0.30 ± 0.05 U/mg prot; HAEC + AD plasma = 0.16 ± 0.03 U/mg prot; HAEC + C plasma + RSV = 0.46 ± 0.008 U/mg prot; HAEC + AD plasma + RSV = 0.25 ± 0.01 U/mg prot. Na^+^/K^+^-ATPase: HAEC + C plasma = 0.578 ± 0.003 µmol P_i_/mg prot/h; HAEC + AD plasma = 0.338 ± 0.05 µmol P_i_/mg prot/h; HAEC + C plasma + RSV = 0.793 ± 0.049 µmol P_i_/mg prot/h; HAEC + AD plasma + RSV = 0.470 ± 0.033 µmol P_i_/mg prot/h; *p* < 0.05) (Figs. **2a** and **2b**, respectively).

**Fig. (3):**
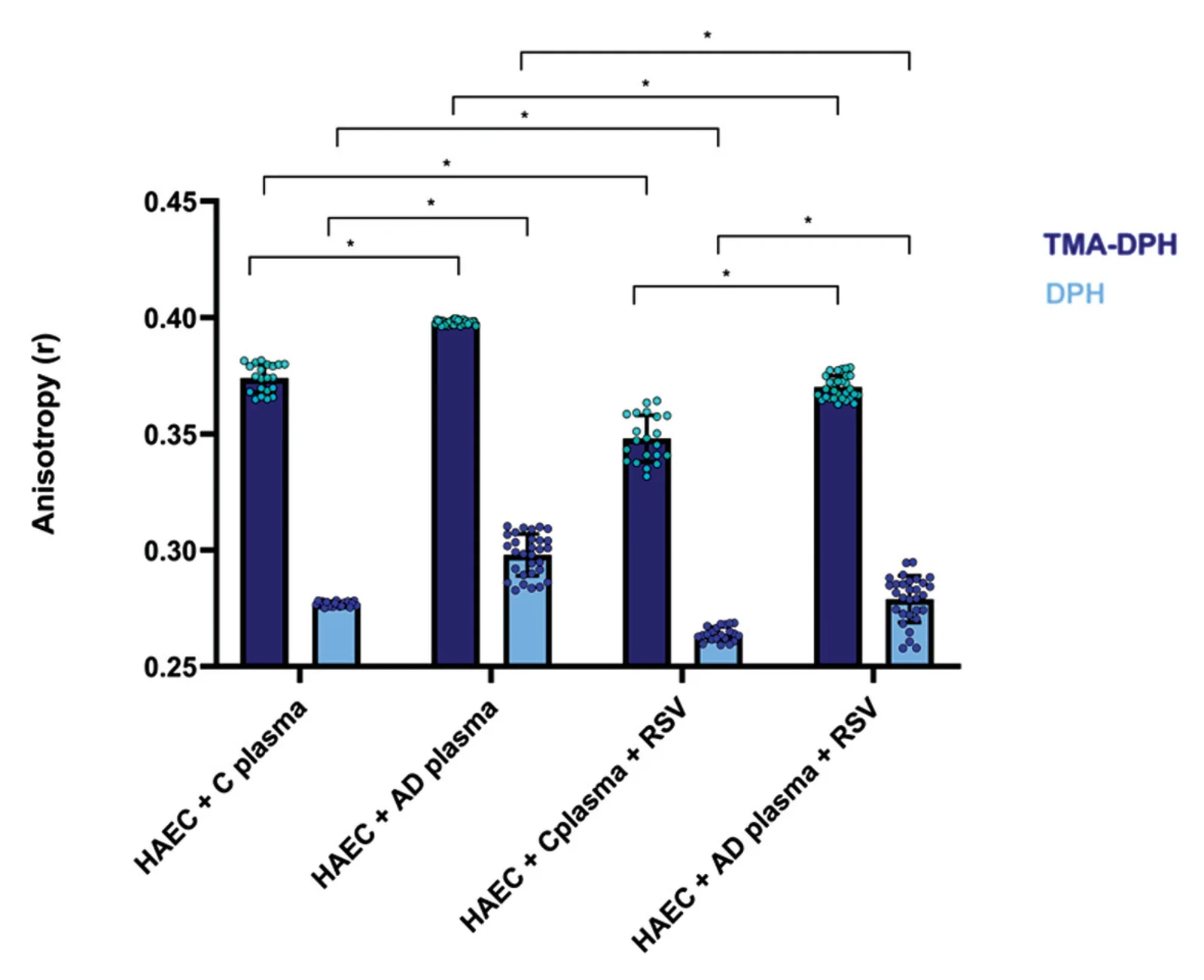
Fluorescence anisotropy of TMA-DPH and DPH in HAECs incubated with control plasma, (HAEC + C plasma), plasma from AD patients (HAEC + AD plasma), plasma from control subjects supplemented with resveratrol (HAEC + C plasma + RSV), and AD plasma supplemented with resveratrol (HAEC + AD plasma + RSV). Means ± S.D. and individual data points are shown. *p < 0.05.

Membrane fluidity was also significantly affected by plasma treatment. Anisotropy values measured with TMA-DPH and DPH probes—reflecting the outer and inner membrane regions, respectively—were significantly increased in cells exposed to AD plasma, compared to those exposed to control plasma. Since anisotropy is inversely related to membrane fluidity, our findings indicate a marked decrease in fluidity in cells treated with AD plasma. In addition, our results evidenced a higher endothelial cell membrane fluidity after the exposure to RSV supplemented plasma (being TMA-DPH: HAEC + C plasma = 0.374 ± 0.006; HAEC + AD plasma = 0.398 ± 0.01; HAEC + C plasma + RSV = 0.348 ± 0.01; HAEC + AD plasma + RSV = 0.370 ± 0.005; DPH: HAEC + C plasma = 0.277 ± 0.001; HAEC + AD plasma = 0.298 ± 0.009; HAEC + C plasma + RSV = 0.264 ± 0.003; HAEC + AD plasma+ RSV = 0.279 ± 0.01; *p* < 0.05) (Fig. **3**).

**Fig. (4):**
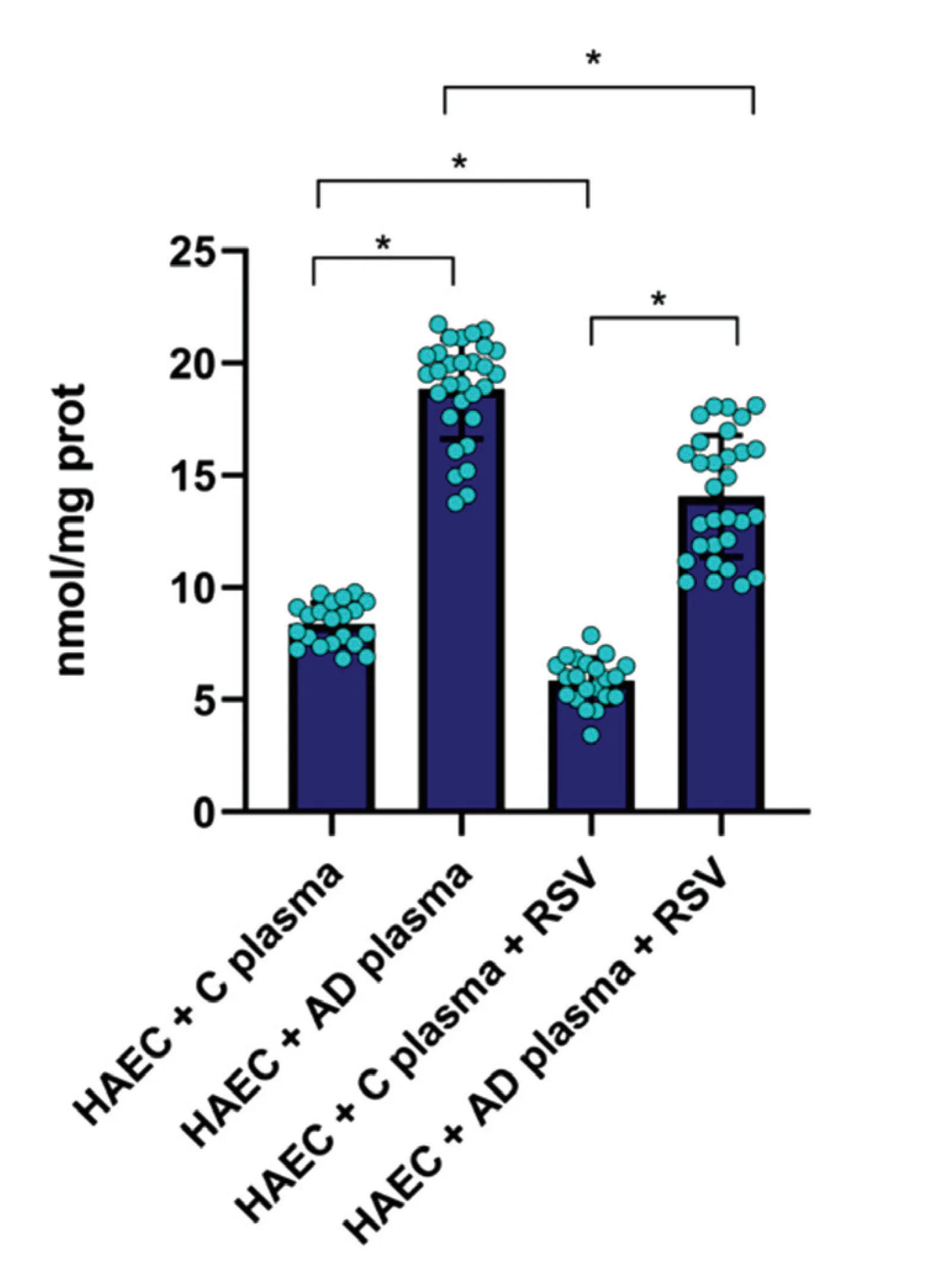
TBARS levels in HAEC following incubation with plasma from healthy subjects (HAEC + C plasma), AD plasma (HAEC + AD plasma), control plasma supplemented with resveratrol (HAEC + C plasma + RSV), and AD plasma supplemented with resveratrol (HAEC + AD plasma + RSV). Means ± S.D. and individual data points are shown. *p < 0.05.

Lastly, lipid peroxidation in HAECs, assessed by TBARS levels, was significantly increased in cells exposed to AD plasma compared to control plasma. However, RSV supplementation caused a notable decrease in TBARS levels in both groups (being HAEC + C plasma = 8.37 ± 0.95 nmol/mg prot; HAEC + AD plasma = 18.85 ± 2.23 nmol/mg prot; HAEC + C plasma + RSV = 5.84 ± 1.03 nmol/mg prot; HAEC + AD plasma + RSV = 14,08 ± 2,71 nmol/mg prot; *p* < 0.05) (Fig. **4**).

## 4 DISCUSSION

The pathogenesis of sporadic AD remains incompletely understood; however, early signs of vascular dysfunction have been consistently observed in AD patients, often preceding Aβ accumulation [31, 32]. It has been suggested that circulating amyloid peptides may have a toxic effect on the endothelium, leading to endothelial-dependent vasoconstriction due to reduced NO levels. This contributes to diminished cerebral perfusion, compounded by factors such as aging and vascular wall thickening. These changes hinder the delivery of nutrients and oxygen to brain cells and impair Aβ clearance [4, 9, 33-36]. Although research into the molecular mechanisms underlying AD is advancing, currently there is no cure for the disease, and only symptomatic treatments are available [37]. The endothelium is a promising therapeutic target due to the reversibility of endothelial dysfunction. Building on previous results, this study investigates the *in vitro* effects of plasma from AD patients and healthy controls on HAECs, before and after supplementation with RSV, a compound known for its antioxidant properties that may help treat diseases with high oxidative damage [20]. We specifically measured NO and peroxynitrite production, SOD and Na^+^/K^+^-ATPase enzymatic activities, membrane fluidity, and TBARS levels. Our results showed a notable decrease in NO levels in HAECs incubated with AD plasma compared to those exposed to control plasma, which aligns with previous findings from our group [18]. This decreased NO production could initiate a damaging feedback loop, accelerating neurodegeneration. However, RSV supplementation significantly increased NO production in both AD and control plasma-incubated cells. This may be due to the stimulation of eNOS expression and activity, as well as the reversal of eNOS uncoupling, as previously suggested [38]. Additionally, RSV's antioxidant properties may enhance NO bioavailability, further boosting NO levels. We detected significantly higher levels of peroxynitrite production in HAECs treated with AD plasma compared to those exposed to control plasma, in line with previous studies showing elevated peroxynitrite levels in AD animal models [39]. After RSV supplementation, peroxynitrite levels decreased in both groups. Similar trends were observed for SOD and Na^+^/K^+^-ATPase activities, with both enzymes showing significant inhibition after exposure to AD plasma compared to cells incubated with control plasma. This supports prior findings from our group and others, showing reduced antioxidant enzyme activity and impaired Na^+^/K^+^-ATPase function in AD patients [18, 39-42]. The reduction in SOD activity increases the endothelium's susceptibility to oxidative damage. The reduced activity of Na^+^/K^+^-ATPase, which is a target for both reactive oxygen species (ROS) and reactive nitrogen species (RNS), may also result from altered plasma membrane fluidity in cells exposed to AD plasma. This reduction in Na^+^/K^+^-ATPase activity can cause osmotic imbalances, potentially leading to cell death [29, 41]. Both SOD and Na^+^/K^+^-ATPase activities were restored following RSV supplementation, which also reduced membrane rigidity in HAECs, consistent with previous studies on diabetic platelets [25]. In terms of lipid peroxidation, measured by TBARS, we found significantly increased levels in cells exposed to AD plasma as compared to those exposed to control plasma. This finding aligns with our previous results and with studies showing elevated lipid peroxidation and nucleic acid oxidation in AD brains, as well as higher oxidative stress biomarkers in MCI and late-onset AD [11, 42, 43]. The overproduction of free radicals, combined with decreased antioxidant enzyme activity, likely contributes for reduced NO bioavailability and endothelial cell membrane fluidity, not only in the brain but also systemically. Prior studies have shown increased membrane rigidity in AD brains, erythrocytes, and platelets [29, 44-46]. TBARS levels were significantly reduced, following RSV supplementation, supporting earlier studies demonstrating RSV's protective effect against lipid peroxidation in cell membranes [47]. In summary, our findings emphasize the positive effects of resveratrol on the vascular endothelium, where it reduces ROS levels, boosts antioxidant enzyme activity, and enhances endothelial function by improving membrane fluidity and Na^+^/K^+^-ATPase activity. Additionally, RSV may help counteract cerebral hypoperfusion by increasing NO production and improving its bioavailability.

## CONCLUSION

In conclusion, our study reinforces the hypothesis that the vascular endothelium plays a pivotal role in the pathophysiology of AD, and that systemic vascular alterations may contribute to the onset and progression of neurodegeneration. By demonstrating that plasma from AD patients induces significant endothelial dysfunction—characterized by oxidative stress, impaired nitric oxide signalling, reduction of enzymatic activities, and altered membrane properties—our findings underscore the importance of targeting vascular health in AD.

Moreover, our data highlight the promising role of RSV, a natural polyphenolic compound, in mitigating these detrimental effects. RSV supplementation effectively improved endothelial cell function by restoring antioxidant enzyme activity, reducing reactive oxygen and nitrogen species, enhancing membrane fluidity, and increasing NO bioavailability. These findings support the potential of RSV and similar bioactive dietary compounds as adjunctive therapeutic agents aimed at reducing oxidative stress and preserving endothelial integrity in AD patients.

Although preliminary and limited by sample size, these results open new avenues for the development of preventive and therapeutic strategies focused on vascular protection. Targeting endothelial dysfunction through safe, naturally occurring compounds may represent a complementary approach to slow disease progression, improve cerebral perfusion, and ultimately delay cognitive decline in Alzheimer’s disease.

## STUDY LIMITATIONS

The main limitation of the present study was the relatively small sample size, which may not fully capture the variability within the broader population and may limit the generalizability of the findings. Future research should aim to recruit a larger sample to validate these initial results and provide more generalizable insights.

## Data Availability

The data and supportive information are available within the article.

## LIST OF ABBREVIATIONS

AD: Alzheimer’s Disease
Aβ: Amyloid β
BBB: Blood-brain Barrier
CNS: Central Nervous System
MCI: Mild Cognitive Impairment
NO: Nitric Oxide
NOS: Nitric Oxide Synthase
RNS: Reactive Nitrogen Species
ROS: Reactive Oxygen Species
TBA: Thiobarbituric Acid

## STANDARDS OF REPORTING

STROBE guidelines were followed.
